# Editorial: Synthesizing the Evidence on Prisoner Health—Taking Stock and Moving Forward

**DOI:** 10.1093/aje/kwx375

**Published:** 2018-04-27

**Authors:** Seena Fazel

**Affiliations:** Department of Psychiatry, University of Oxford, Warneford Hospital, Oxford United Kingdom

More important than the fact that 10 million people are currently estimated to be in prison at any point is that approximately 30 million persons enter and leave prisons every year (1). The public health opportunities arising from these large numbers have been outlined in many articles in the most recent issue of *Epidemiologic Reviews*; namely, that individuals entering correctional settings are typically from marginalized groups who do not access health care in the community and who have a high prevalence of many communicable and noncommunicable diseases and incidence of risky behaviors. Furthermore, being incarcerated increases the risks of morbidity and mortality, especially from conditions secondary to infectious diseases and substance misuse, and thus prison affords an important opportunity to address unmet health-care needs with substantial consequences for public health (2, 3).

The current issue of *Epidemiologic Reviews* comprises a number of systematic reviews and meta-analyses in which the authors address emerging challenges for prison health—delineating the health problems of the growing number of older prisoners (4), understanding the adverse effects of incarceration on the families of those in custody (5), and mitigating risky behaviors in prison (6, 7)—and synthesize the latest evidence on health problems that remain central in prison, such as the prevalence of treatable mental disorders (8), interventions for and the modeling of infectious diseases (9), treatments for substance misuse (10), and health needs of prisoners in low- and middle-income countries (11). The 2 overriding themes that are apparent from reading these papers are 1) the methodological issues that cut across the different reviews and 2) the various implications arising from them.

Many of the systematic reviews in this issue follow methodological guidelines (such as the Preferred Reporting Items for Systematic Reviews and Meta-Analyses) and utilize excellent methods. In particular, it is important that gray literature has been searched, because many relevant studies are published as reports rather than in peer-reviewed articles, and that there has been correspondence with the authors of the primary studies to clarify results. Furthermore, authors of some reviews preregistered their protocols (8). In a number of reviews, the search for primary articles and reports was limited by country or region. This is reasonable when clinical heterogeneity is substantial and can be informative by focusing on clinical, research, and policy implications specific to the country or region. Hence, in the review on substance use in low- and middle-income countries (11), investigators reported differences by subregions and also focused on substance use rather than diagnostic categories because of the nature of the data collected in the primary studies. In another review, the authors studied the increasingly important question of the health of older prisoners and restricted their search to US studies (4). Despite this restriction, 21 studies were identified, and, reflecting the review’s focus, there was a discussion of US national policies. At the same time, this US-centric approach meant that the review did not draw on high-quality research in other high-income countries, which have similar proportions of incarceration by age group (12, 13). For example, Skarupski et al. (4) found no research on cognitive problems, although there has been recent relevant research on cognitive impairment from a large sample in the United Kingdom (12). Some of the papers, however, included more individual approaches. One inclusion criterion used by Wildeman et al. was that a primary study should have been published in certain “peer-reviewed journals that are well-regarded in their respective fields” (5, p. 148). Arguably, this breaks with one of the key features of a systematic review (as compared with a narrative review), which aims to provide a comprehensive and transparent selection process. Many reviews were limited to English-language articles, which may have led the authors to miss important negative studies because these are more likely to be published in non–English-language journals. Another issue that is that some of the systematic reviews contained a small number of studies, with one including 5 primary studies and another including 7 publications. This is not in itself problematic because it can highlight the need for more research and new recommendations. Nevertheless, a scoping review might have identified the likely small number of primary reports, and consideration could have been given to broadening the aims of the review. A final issue that cuts across the reviews is the time period utilized in the article search. One of the reviews is a 21-month update (which extends the search for primary reports from November 2015 to July 2017), and the authors identified 9 new papers to bring the total to an impressive 82 primary reports (14). Methodologists have considered what constitutes an update for a systematic review, which could be undertaken when there is significant new work in a particular field (15) or a doubling of the available evidence, although treatment reviews may require more regular updates (such as required by the Cochrane Collaboration). Finally, the review by Spaulding et al. (7) on smoking in prisons is outstanding on many levels. It has a broad and overlapping aims on prevalence and interventions. The methods are also excellent, including a specific search in a Chinese database, which some have argued should be included for any global systematic review on prevalence of common conditions (16). The findings are unexpected, informative, and highly relevant, and the importance of the topic is underscored and complemented by another paper in the issue on substance use in prisoners in low- and middle-income countries, in which investigators found high rates of smoking inside custody (11).

The papers usefully raise many implications for clinicians, researchers, and policy makers. First, they provide more precise information on the prevalence of infectious diseases, posttraumatic stress disorder, substance misuse, and, in older US prisoners, a range of noncommunicable diseases. Interestingly, the prevalence estimates for posttraumatic stress disorder among female prisoners in the review by Mundt et al. (8) are at the upper end of the range from a previous 2007 systematic review (17). Second, there is new information on risky behaviors in prison, from smoking to tattooing, piercing, engaging in sexual activities, and syringe sharing (6). Moazen et al. (6) highlighted the lack of evidence-based infection control measures to address these risks; these measures are important for public health and are likely to be cost-effective. Third, the review on dynamic models of the spread of infectious disease in prison is novel, raises helpful methodological pointers, and might interest researchers estimating the direct and indirect effects of interventions for other diseases (9). Finally, there are a number of systematic reviews in the current issue in which the authors evaluated treatments. Despite not including pooled treatment effect sizes, the review on drug and alcohol interventions is of considerable interest and presents information from 49 studies (10). In the review, the authors used a quality rating instrument and included observational studies, which can be informative for treatments for which randomized controlled trials are not feasible in prison settings. In keeping with this, large population-based observational studies have been used to study pharmacological treatments to prevent violent reoffending in released prisoners, whereas randomized controlled trials have been lacking (18). However, findings from these designs need to carefully consider confounding by indication, and approaches such as within-individual designs are increasingly used to account for confounding (18, 19). In their systematic review, de Andrade et al. (10) found evidence in support of opiate maintenance treatment; unexpectedly, they also reported that cognitive behavioral therapy did not reduce recidivism outcomes and that its effects on substance use were limited. This is important because cognitive behavioral therapy is widely used in prisons, and their findings are consistent with research on psychological treatments for common mental health disorders that suggest weaker-than-expected efficacy for such interventions (20).

In summary, this collection of articles highlights the public health challenges and opportunities in prison health, fills some important gaps in the correctional health literature, and provides many thoughtful suggestions for future research. Such research should aim to study unselected prison populations and consider utilizing innovative designs to overcome the challenges of conducting research in prisons. Looking forward to new systematic reviews in prison health, researchers should include gray literature and non–English-language articles, consider protocol registration, and explore heterogeneity carefully using subgroup and metaregression analyses. Other considerations for such reviews include checking with authors of primary studies whether their data is accurately presented (when possible) and sharing summary data in published supplemental materials (21). Improvements to prison health will be informed by higher quality rather than a greater quantity of systematic reviews and meta-analyses.
